# Isolation of diverse members of the *Aquificales* from geothermal springs in Tengchong, China

**DOI:** 10.3389/fmicb.2015.00157

**Published:** 2015-02-27

**Authors:** Brian P. Hedlund, Anna-Louise Reysenbach, Liuquin Huang, John C. Ong, Zizhang Liu, Jeremy A. Dodsworth, Reham Ahmed, Amanda J. Williams, Brandon R. Briggs, Yitai Liu, Weiguo Hou, Hailiang Dong

**Affiliations:** ^1^School of Life Sciences, University of NevadaLas Vegas, Las Vegas, NV, USA; ^2^Nevada Institute of Personalized Medicine, University of NevadaLas Vegas, Las Vegas, NV, USA; ^3^Biology Department and Center for Life in Extreme Environments, Portland State UniversityPortland, OR, USA; ^4^State Key Laboratory of Biogeology and Environmental Geology, China University of GeosciencesBeijing, China; ^5^Department of Biology, California State University San BernardinoSan Bernardino, CA, USA; ^6^Department of Geology and Environmental Earth Science, Miami University,Oxford, OH, USA

**Keywords:** *Aquificales*, *Hydrogenobacter*, *Hydrogenobaculum*, *Sulfurihydrogenibium*, hot springs, hydrogen oxidation, sulfide oxidation, thiosulfate oxidation

## Abstract

The order *Aquificales* (phylum *Aquificae*) consists of thermophilic and hyperthermophilic bacteria that are prominent in many geothermal systems, including those in Tengchong, Yunnan Province, China. However, *Aquificales* have not previously been isolated from Tengchong. We isolated five strains of *Aquificales* from diverse springs (temperature 45.2–83.3°C and pH 2.6–9.1) in the Rehai Geothermal Field from sites in which *Aquificales* were abundant. Phylogenetic analysis showed that four of the strains belong to the genera *Hydrogenobacter*, *Hydrogenobaculum*, and *Sulfurihydrogenibium*, including strains distant enough to likely justify new species of *Hydrogenobacter* and *Hydrogenobaculum*. The additional strain may represent a new genus in the *Hydrogenothermaceae*. All strains were capable of aerobic respiration under microaerophilic conditions; however, they had variable capacity for chemolithotrophic oxidation of hydrogen and sulfur compounds and nitrate reduction.

## INTRODUCTION

The phylum *Aquificae* is composed of a single order, *Aquificales*, and three families, *Aquificaceae*, *Hydrogenothermaceae*, and *Desulfurobacteriaceae* (Reysenbach et al., 2005; L’Haridon et al., 2006). *Aquificales* are present in many terrestrial and marine geothermal systems where they often form multicellular “streamer” assemblages (Huber et al., 1998; Reysenbach et al., 2000, 2005; Takacs et al., 2001; Eder and Huber, 2002; Spear et al., 2005; Hou et al., 2013; Takacs-Vesbach et al., 2013) but can also be prominent members of planktonic microbial communities (Cole et al., 2013; Hou et al., 2013; Murphy et al., 2013). Most members of the *Aquificales* are obligate or facultative autotrophs (Kawasumi et al., 1984; Stohr et al., 2001; Takai et al., 2001; Eder and Huber, 2002; Aguiar et al., 2004; Caldwell et al., 2010), although at least one isolate was reported to be incapable of autotrophic growth under the conditions that were tested (Takai et al., 2001). Although very few studies have quantified autotrophy in terrestrial geothermal systems inhabited by *Aquificales* (Boyd et al., 2009), *Aquificales* are broadly hypothesized to be important primary producers and are capable of using a variety of inorganic compounds to fuel chemolithotrophy, including diverse electron donors (H_2_, S^2-^, S_2_O_3_^2-^, SO_3_^2-^, S^0^, Fe^2+^, AsO_3_^3-^) and terminal electron acceptors (O_2_, NO_3_^-^, SO_3_^2-^, Fe^3+^, AsO_4_^3-^, SeO_3_^2-^; Stohr et al., 2001; Takai et al., 2001; Eder and Huber, 2002; O’Neill et al., 2008).

Two families of *Aquificales* dominate in terrestrial geothermal systems, the *Aquificaceae* and *Hydrogenothermaceae*. The *Aquificaceae* includes three genera that are abundant in terrestrial systems: *Hydrogenobacter*, *Thermocrinis*, and *Hydrogenobaculum* (Reysenbach, 2001; Takacs-Vesbach et al., 2013). *Hydrogenobacter* and *Thermocrinis* are closely related and are capable of axenic growth at circumneutral pH to ≥85°C (Kawasumi et al., 1984; Takai et al., 2001; Eder and Huber, 2002) and ≥89°C (Huber et al., 1998; Eder and Huber, 2002; Caldwell et al., 2010), respectively. In contrast, known isolates of *Hydrogenobaculum* are acidophilic (optimum pH 3–4) and have lower growth temperature ranges, with optima between 60 and 70°C (Shima and Suzuki, 1993; D’Imperio et al., 2008). The family *Hydrogenothermaceae* includes a single genus that is prominent in many terrestrial geothermal systems, *Sulfurihydrogenibium*, with known isolates capable of growth to ≥75°C at circumneutral pH (5.0–8.8; O’Neill et al., 2008).

**FIGURE 1:**
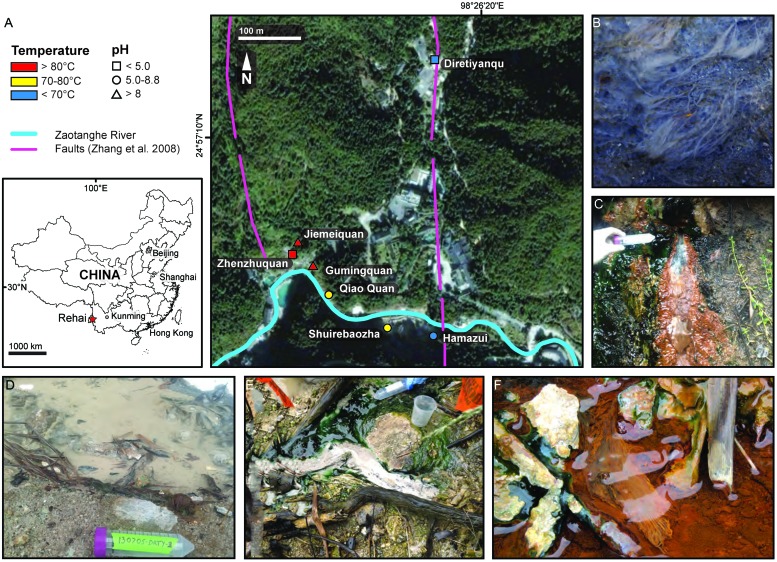
**Study area map and sample locations.** Strains were isolated from five hot spring locations in the Rehai Geothermal Field **(A)** in Yunnan, China: **(B)** *Hydrogenobacter* sp. T-2 from a white streamer community in Gumingquan (Drum Beating Spring) pool site Gmq-P (82.9°C, pH 8.94), **(C)** *Hydrogenobacter* sp. T-8 from Qiao Quan (Bridge Spring) site QQ (74.6°C, pH 6.36), **(D)** *Hydrogenobaculum* sp. T-6 from Diretiyanqu (Experimental) site DRTY (60.0°C, pH 2.62), **(E)** *Hydrogenothermaceae* strain T-5 from a white/yellow streamer community in a sulfurous seep on a hillside south of Shuirebaoza, site Srbz-U (70.0°C, pH 6.6), **(F)** *Sulfurihydrogenibium subterraneum* T-7 from a white/yellow streamer community in Hamazui-3 (spring located ∼5 m SE of Hamazui), site HMZFJ-3 (68.0°C, pH 6.50). **(A)** is modified from Hedlund et al. (2012). Fifty mL conical tubes used for scale are 11.7 cm long.

Yunnan Province, in southwest China, has a large number of geothermal springs, particularly in Tengchong County, which is located within the Indo-Burma Range along the central-western border between Yunnan Province and Myanmar. Geothermal activity in Yunnan Province is typically located along arched fault structures and circular depressions and is likely fueled by latent heat from tectonic activity associated with the subduction of Tethys Ocean lithosphere (Liao and Guo, 1986; Wang et al., 2008). The largest and best-known geothermal area in Tengchong is the Rehai (“Hot Sea”) Geothermal Field, with springs reaching the boiling point (∼95°C at ∼1,500 m elevation) and spanning a pH range of 2.5–9.4 at high temperature (>80°C; **Figure 1**; **Table 1**; Hedlund et al., 2012). A large number of Bacteria and Archaea have been isolated from Rehai springs, particularly thermophilic members of the *Firmicutes* (*Bacillales*, *Thermoanaerobales*, *Clostritiales*), *Deinococcus-Thermus* phylum (*Thermales*), and *Crenarchaeota* (*Sulfolobales*) (reviewed in Hedlund et al., 2012). However, despite recent cultivation-independent studies suggesting that *Aquificales* are abundant in nearly all high-temperature sites in Rehai (Pagaling et al., 2012; Hou et al., 2013; Song et al., 2013; Briggs et al., 2014), there are no published reports of the isolation or characterization of *Aquificales* from Rehai or anywhere in China.

**Table 1:** Sources of *Aquificales* strains isolated from Tengchong hot springs and their 16S rRNA gene sequences.

Organism*	Source location and characteristics	Accession numbers
*Hydrogenobacter* sp. T -2	White streamer community in Gumingquan (Drum Beating Spring) pool near site Gmq-P (82.9°C, pH 8.94; GPS N24.95093°, E98.43626°)	KP175576
*Hydrogenobacter* sp. T -8	Small white streamers above iron oxide mat in Qiao Quan (Bridge Spring) site QQ (74.6°C, pH 6.36; GPS N24.95044°, E98.43650°)	KP175579
*Hydrogenobaculum* sp. T -6	Bulk sediment and water Diretiyanqu (Experimental) site DRTY (60.0°C, pH 2.62; GPS N24.95390°, E98.43819°)	KP125885
*Hydrogenothermaceae* strain T -5	White streamer community in sulfurous seep on hillside southwest of Shuirebaoza, site Srbz-U (70.0°C, pH 6.6; GPS N24.95002°, E98.43743°)	KP175577
*S. subterraneum* T -7	Iron oxide mat/streamer community near Hamazui (spring located ∼5 m SE of Hamazui) site HMZFJ-1 (68.0°C, pH 6.50; GPS N24.94992°, E98.43808°)	KP175578

*Samples for T-2, T-5, T-7, and T-8 were collected on 07/04/13. The sample for T-6 was collected on 12/29/12. See ***Figure 1*** for photos of sampling sites.

In this study, we isolated *Aquificales* from sites in Tengchong known to host abundant *Aquificales* populations and sites with abundant streamer growth that were deemed likely to host *Aquificales*. The strains belong to the genera *Hydrogenobacter*, *Hydrogenobaculum*, and *Sulfurihydrogenibium*, and possibly a new genus within the *Hydrogenothermaceae*. Although most of the strains likely represent new taxa, their general physiological traits are similar to known members of these genera, including variable capacity for aerobic hydrogen oxidation via the “knallgas reaction,” chemolithotrophic oxidation of sulfur compounds, and anaerobic respiration of nitrate.

## MATERIALS AND METHODS

### SAMPLE COLLECTION, ENRICHMENT, AND ISOLATION

Sediment, streamer, and mat samples were collected from five hot springs located in the Rehai Geothermal Field in Tengchong County, Yunnan Province, China (**Figure 1**). Prior to sampling, the temperature and pH were measured at the precise sampling location with a field-calibrated pH probe with temperature correction (LaMotte five Series, Chestertown, MD, USA). Detailed water chemistry and microbial community composition at most of the sampling locations on several previous sampling trips has been reported elsewhere (Hou et al., 2013; Briggs et al., 2014).

Samples from which strains T-2, T-5, T-7, and T-8 were isolated were collected aseptically and transferred into 25 mL Balch tubes containing 5 mL modified MSH medium (Caldwell et al., 2010) containing S^0^ and S_2_O_3_^2-^ and adjusted to pH 8.0 (T-2), 6.5 (T-5 and T-7), or 7.5 (T-8). Tube headspace was either H_2_:CO_2_ (80:20) for strains T-2, T-7, and T-8, or N_2_:CO_2_ (80:20) for strain T-5, supplemented with 4% v/v O_2_. The tubes were stored and transported at room temperature. Once in the lab, the Balch tubes were incubated at 80°C (T-2) or 70°C (T-5, T-7, and T-8) and passaged in the same medium under the same conditions. To obtain pure cultures of strains T-2, T-7, and T-8, positive enrichments were streaked for isolation onto plates containing GBS salts medium (Dodsworth et al., 2014) containing thiosulfate (1 mM added as Na_2_S_2_O_3_⋅5H_2_O) and solidified with Gelrite [0.8% mass/vol, supplemented with 4 g/L MgCl_2_⋅6H_2_O (Serva, Heidelberg)] and incubated at 70°C in modified two quart Bandit pressure pots (C.A. Technologies). Pressure pot headspace consisted of ∼2 L anaerobic chamber gas (N_2_:CO_2_:H_2_ at 90:5:5) supplemented with 200 mL H_2_:CO_2_ (80:20) and 100 mL air. Isolated colonies were re-streaked two times to ensure purity. Strain T-5 was isolated using an extinction-to-dilution method that was repeated at least seven times. For all strains, purity was confirmed through microscopic observation and sequencing of the 16S rRNA gene, following the general approach described previously (Nakagawa et al., 2005).

The sample from which strain T-6 was isolated was aseptically transferred in the field into a 25 ml Balch tube containing 10 mL of DSMZ medium 743 (modified by replacing S^0^ with 30 μM Na_2_S, pH 3), given a headspace of N_2_/CO_2_/H_2_/air (30:40:20:10), and incubated in the spring. Following growth, the tube was transported to the lab without temperature control. For isolation, 1 mL of the enrichment culture was inoculated into 10 ml of the same medium with the same headspace as in initial enrichment. A pure isolate was obtained by three rounds of dilution to extinction and verified through microscopic observation and sequencing of the 16S rRNA gene.

### GROWTH CHARACTERISTICS

The capacity for growth of the strains on electron donors and electron acceptors commonly used by *Aquificales* was determined by growing each strain under conditions that permitted good growth, as determined by phase-contrast microscopy. In all cases, growth was determined by direct cell counts using a Petroff–Hausser counting chamber and a phase-contrast microscope. All experiments were performed in triplicate along with positive and negative controls. Strains T-2, T-7, and T-8 were routinely grown at 70°C in 5 mL volumes of GBS salts medium (Dodsworth et al., 2014) with an N_2_/H_2_/CO_2_/air (75:17:4:4) headspace or in 25 mL Balch tubes without shaking. The medium was adjusted to pH 8.0, 7.2, and 6.6 for T-2, T-7, and T-8, respectively. Strain T-5 was routinely grown in a modified MSH medium (Caldwell et al., 2010) containing S^0^ and S_2_O_3_^2-^ at 70°C in 5 mL volumes with a headspace of N_2_:CO_2_ (80:20). For testing electron donors, H_2_ was replaced with N_2_ in the headspace (for T-2, T-7, and T-8) and the following compounds were added as sources of possible electron donors, each tested at 1 mM final concentration: Na_2_S_2_O_3_⋅5H_2_O, sodium pyruvate, sodium formate, and sodium acetate; additionally, S^0^ was tested at 0.1 and 1.0% (w/vol). For testing terminal electron acceptors, air was replaced with N_2_ and the following possible electron acceptors were tested at 1 mM final concentration: NaNO_3_, NaNO_2_, Na_2_S_2_O_3_⋅5H_2_O, and Na_2_HAsO_4_.

Strain T-6 was routinely grown at 60°C in 10 mL volume of a modified DSMZ 743 medium with a N_2_/CO_2_/H_2_/air (30:40:20:10) headspace in 25 mL Balch tubes with no shaking. The following compounds were tested as possible electron donors under aerobic conditions with 5 mM citric acid as a buffer (pH 3.0; D’Imperio et al., 2008): S^0^ (w/vol 0.1%), Na_2_S (3 mM), Na_2_S_2_O_3_⋅5H_2_O (100 μM), sodium lactate (1 g/L), sodium pyruvate (1 g/L), sodium formate (1 g/L), and sodium acetate (1 g/L; Shima and Suzuki, 1993). The following compounds were tested as possible terminal electron acceptors in the same medium with H_2_ as the electron donor with an atmosphere of N_2_/CO_2_/H_2_ (40:40:20): NaNO_3_ (100 μM), NaNO_2_ (100 μM), Na_2_S_2_O_3_⋅5H_2_O (100 μM), FeCl_3_ (100 μM), and Na_2_SO_4_ (100 μM; Shima and Suzuki, 1993).

### IDENTIFICATION OF NITRATE REDUCTION PRODUCTS

Nitrate and nitrite were measured colorimetrically using reagents from LaMotte (LaMotte, Chesterton, MD, USA). Nitrate plus nitrite was determined by cadmium reduction of nitrate and subsequent diazotization of nitrite. Nitrite was determined by diazotization without reduction of nitrate. Nitrous oxide was measured by gas chromatography-electron capture detection on a GC-2014 Nitrous Oxide Analyzer (Shimadzu, Moorpark, CA, USA), modified and operated as described (Dodsworth et al., 2011). Production of N_2_ was tested by using Durham vials.

### 16S rRNA GENE PCR, SEQUENCING, AND PHYLOGENETIC ANALYSIS

DNA was extracted using the FastDNA Spin Kit for Soil (MP Biomedicals, Solon, OH, USA) and 16S rRNA genes were amplified by PCR using primers 9 bF (Eder et al., 1999) and 1512uR (Eder et al., 2001) and sequenced as previously described (Costa et al., 2009). Reads were trimmed to remove bases with quality scores of less than 20 and aligned against the SILVA alignment in the program mothur v1.20.2 (Schloss et al., 2009; Quast et al., 2013). Near full-length 16S rRNA gene sequences were aligned along with reference sequences of *Aquificales*, including the closest BLASTN matches, using the SILVA alignment in mothur v1.33.3. The alignment was curated manually using Bioedit v7.0.5.3 (Hall, 1999) and analyzed with and without the Lane mask (Lane, 1991) using maximum likelihood in RAxML v7.2.6 (100 replicates, GTR+CAT model of nucleotide substitution; Stamatakis, 2006) and neighbor joining in PHYLIP v3.69 (1,000 replicates; Felsenstein, 2005). Trees were visualized and manipulated using Dendroscope v2 (Huson et al., 2007). Distances shown in **Table 2** were derived by applying the dist.seqs to the curated Silva alignment. Pairwise comparisons between 16S rRNA gene sequences from isolates and previously published sequence tags (Hou et al., 2013) were computed using DNAMAN software (Lynnon LLC, San Ramon, CA, USA).

**Table 2:** 16S rRNA gene identity to closest cultivated relatives.

Organism	Closest cultivated relative	% Identity	Accession numbers
*Hydrogenobacter* sp. T-2	*“Hydrogenobacter subterraneus”* HGP1^T^	96.6	NR_024729.1
*Hydrogenobacter* sp. T-8	*“H. subterraneus”* HGP1^T^	97.2	NR_024729.1
*Hydrogenobaculum* sp. T-6	*Hydrogenobaculum* sp. Y04AAS1	95.3	CP001130.1
*Hydrogenothermaceae* strain T-5	*S. rodmanii* UZ3-5^T^	94.6	NR_042515.1
*S. subterraneum* T-7	*S. subterraneum* HGMK-1^T^	99.4	NR_036883.1

### NUCLEOTIDE ACCESSION NUMBERS

Near full-length 16S rRNA gene sequences have been deposited in GenBank with the following accession numbers: KP125885 and KP175576–KP175579.

## RESULTS

### ISOLATION AND PHYLOGENETIC ANALYSIS

Chemolithotrophic isolates were obtained from five geochemically diverse sites in the Rehai Geothermal field (**Table 1**), which were chosen based on previous reports of *Aquificales* in Rehai (Hou et al., 2013; Song et al., 2013; Briggs et al., 2014) and identification of additional streamer communities deemed likely to host *Aquificales*. Sample sites included white streamer material and sediment collected from a high pH, high temperature site (**Figure 1B**), a small geothermal seep hosting small white streamers (**Figure 1C**), sediments in a small acidic pool dominated by silicate sand (**Figure 1D**; Hou et al., 2013; Briggs et al., 2014), a white streamer community in a sulfurous seep (**Figure 1E**), and a biofilm and streamer community encrusted with iron oxide (**Figure 1F**).

**FIGURE 2:**
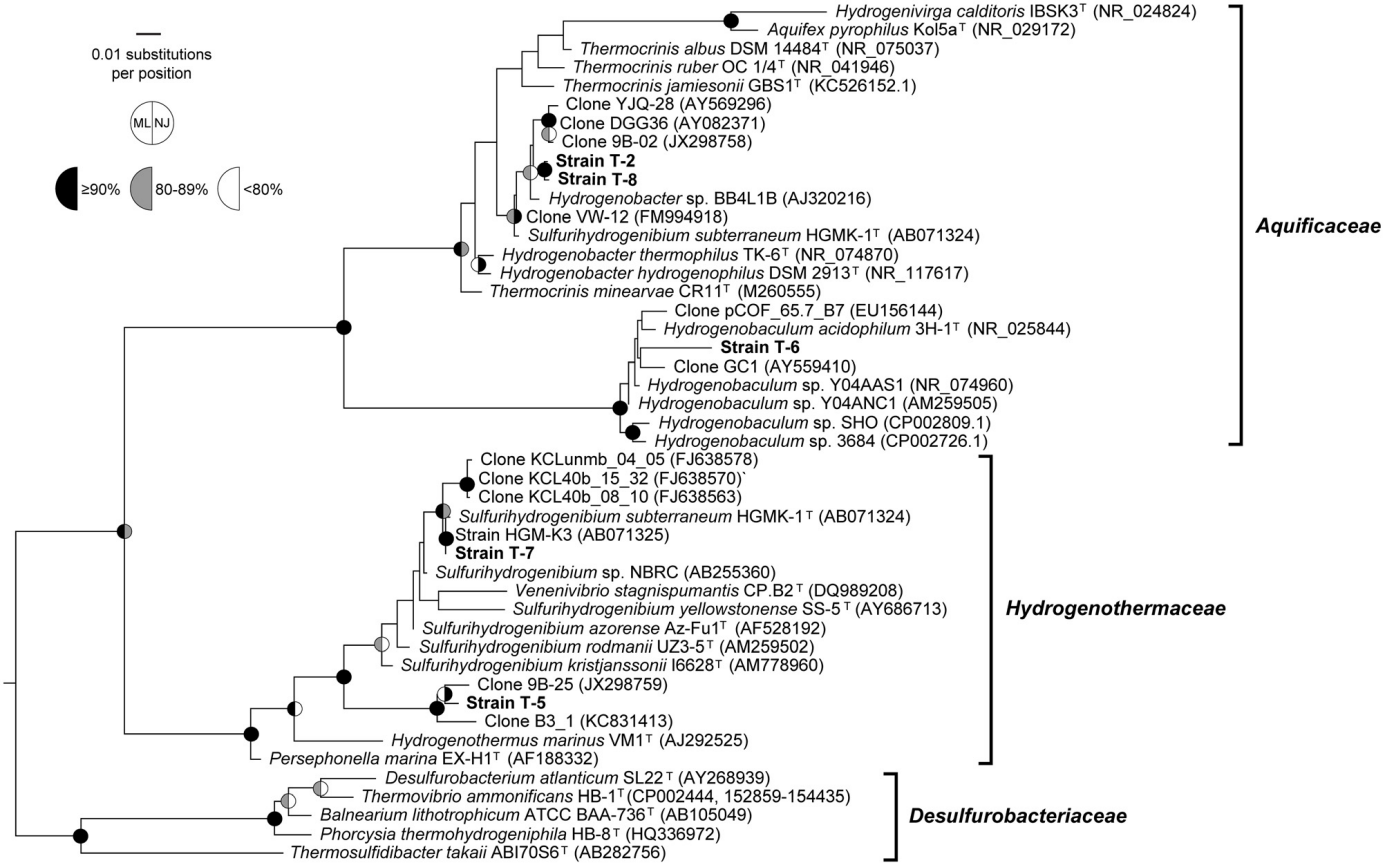
**Phylogenetic analysis.** Maximum-likelihood (ML) phylogeny of the *Aquificales* including all genera and type strains of all species in the genera *Hydrogenobacter*, *Hydrogenobaculum*, and *Sulfurihydrogenibium*, as well as closely related clones from cultivation-independent studies. Bootstrap values represent 100 replicates for ML and 1,000 replicates for neighbor joining (NJ). Similar analyses with a Lane mask or without an outgroup sequence yielded similar results. Bootstrap support for nodes supported by<80% recovery from both methods is not shown. Bar, 0.01 changes per nucleotide. The outgroup was *Methanocaldococcus jannaschii* (AB603516).

Phylogenetic analysis based on near-complete 16S rRNA genes showed that the strains belonged to the families *Aquificaceae* and *Hydrogenothemaceae*. Two *Hydrogenobacter* strains were isolated, designated T-2 and T-8, from sites differing in pH by > 2.5 units. They were grown in media with pH similar to their environmental source, although both were closely related to “*Hydrogenobacter subterraneus*” (**Table 2**; **Figure 2**). Both strains belonged to a species-level (98.65% identity; Kim et al., 2014) operational taxonomic unit (OTU) that comprised >50% of 16S rRNA gene sequence tags in either sediments or the bulk water in most circumneutral geothermal sites in both Rehai and Ruidian (Dientan) geothermal fields (Hou et al., 2013), including streamer and sediment communities in Guminquan, from which strain T-2 was isolated. However, the most abundant sequence within that OTU shared only 98.84% identity with strains T-2 and T-8, whereas the identical sequence to T-2 and T-8 was a rare variant in the cultivation-independent datasets. The other isolate belonging to the *Aquificaceae*, strain T-6, was isolated from the acidic pool, Diretiyanqu. Strain T-6 branched within the genus *Hydrogenobaculum* but was distant from the only validly described species, *Hydrogenobaculum acidophilus*, as well as other isolates from Yellowstone National Park (**Table 2**; **Figure 2**). Strain T-6 belonged to an OTU that comprised 31 to 66% of 16S rRNA gene sequence tags from pools ranging from 55 to 65°C from Diretiyanqu, the system of small acidic pools from which the strain was isolated (Hou et al., 2013). Within these systems, the dominant OTU was identical to T-6. *Sulfurihydrogenibium* strain T-7 was closely related to *Sulfurihydrogenibium subterraneum* and tentatively identified as a member of that species. Within the genus *Sulfurihydrogenibium*, *Sulfurihydrogenibium subterraneum* HGMK-1^T^, and strain T-7 branched with 16S rRNA gene clones from Asia (Japan and Taiwan), potentially representing a species exclusive to Asia (**Figure 2**). T-5, the other strain that branched from within the *Hydrogenothermaceae*, was only distantly related to cultivated strains of *Sulfurihydrogenibium* and *Venenivibrio* and branched with 16S rRNA gene clones from hot springs in China and Thailand (**Table 2**; **Figure 2**; JX298759 and KC831413, unpublished). Aside from strain T-7, the 16S rRNA gene identity between each new isolate and the most closely related species was well below the 16S rRNA gene identity threshold suggested to delimit bacterial species (98.65%; Kim et al., 2014), and strains T-6 and T-5 were also below the median 16S rRNA gene identity circumscribing bacterial genera (Yarza et al., 2014). Formal taxonomic treatment of these isolates will be determined pending detailed physiological and genomic analysis.

With the exception of T-5, all strains were capable of chemotrophic growth with H_2_ as the electron donor under microaerophilic conditions (**Table 3**). Both *Hydrogenobacter* strains also used S_2_O_3_^2-^ as an electron donor and *Hydrogenobacter* sp. T-2 additionally used S^0^ and acetate as electron donors. *Hydrogenobacter* sp. T-8 grew anaerobically by reducing nitrate. Neither nitrous oxide nor dinitrogen were identified as products of nitrate reduction. *Hydrogenobaculum* strain T-6 was capable of microaerobic growth with S^2-^ and S^0^ as alternative electron donors. *S. subterraneum* T-7 was capable of growth with S^2-^ and S_2_O_3_^2-^ as alternative electron donors. T-5 could only use sulfur or thiosulfate as electron donors and O_2_ as the electron acceptor. All strains could grow autotrophically, with the exception of *Hydrogenobaculum* strain T-6, which required or was greatly stimulated by citrate, which is the buffer for DSM medium 743.

**Table 3:** Media for routine growth and growth characteristics for *Aquificales* strains from Tengchong hot springs.

Organism	Medium for routine growth (gas phase vol.)	Temperature (°C)	pH	Electron donors*	Electron acceptors
*Hydrogenobacter* sp. T -2	GBS salts medium (N_2_/H_2_/CO_2_/ air; 75:17:4:4)	70	8.0	H_2_, S_2_O_3_^2-^, S^0^, acetate	O_2_
*Hydrogenobacter* sp. T -8	GBS salts medium (N_2_/H_2_/CO_2_/air; 75:17:4:4)	70	6.6	H_2_, S_2_O_3_^2-^	O_2_, NO_3_^-^
*Hydrogenobaculum* sp. T -6	DSMZ 743 medium(N_2_/CO_2_/H_2_/air; 30:40:20:10)	60	3.0	H_2_, S^2-^, S^0^	O_2_
*Hydrogenothermaceae* strain T -5	Modified MSH medium (CO_2_/O_2_; 76:4)	70	6.5	S_2_O_3_^2-^, S^0^	O_2_
*S. subterraneum* T -7	GBS salts medium (N_2_/H_2_/CO_2_/ air; 75:17:4:4)	70	7.2	H_2_, S_2_O_3_^2-^, S^0^	O_2_

*Electron donors and acceptors that yielded positive growth, defined as a mean cell count of *>5.0 × 10^* 5*^* cells/mL for triplicate growth experiments. All growth experiments were conducted in tandem with triplicate positive and negative controls.

## DISCUSSION

*Aquificales* are globally distributed and often abundant in both marine and terrestrial geothermal systems where they likely play important roles in C, N, H, and S cycles. Recent cultivation-independent censuses of Bacteria and Archaea in hot springs in Tengchong County, China suggested the wide distribution of *Aquificales* in the region, particularly in the Rehai Geothermal System, where *Aquificales* dominated many 16S rRNA gene pyrotag datasets generated using a few different primer sets and on several different sampling campaigns (Pagaling et al., 2012; Hou et al., 2013; Song et al., 2013). Both 16S rRNA gene pyrotag data and phylochip data suggest that *Hydrogenobacter* is a dominant member of most circumneutral to alkaline springs in Rehai (pH 8.1–9.4; Hou et al., 2013; Song et al., 2013; Briggs et al., 2014), including large growths of white streamer material in springs Gumingquan and Jiemeiquan (**Figure 1A**; Hou et al., 2013; Briggs et al., 2014). Strains T-2 and T-8 shared 98.84% 16S rRNA gene sequence identity across the V4 region, suggesting they may belong to the same species as the dominant *Hydrogenobacter* OTU in the springs. However, extrapolation of physiological traits of these strains to the abundant natural populations must be done with caution, since even T-2 and T-8 were different with regard to both electron donor and acceptor use, despite being nearly identical across the near-complete 16S rRNA gene. High intra-species variation in respiratory capacity may be a common feature in the *Aquificales* (D’Imperio et al., 2008). The electron donors and acceptors used by the *Hydrogenobacter* isolates are similar to those described for other members of the genus (Kawasumi et al., 1984; Takai et al., 2001; Eder and Huber, 2002). *“H. subterraneus,”* the most closely related isolate described in detail, is similar in its ability to use reduced sulfur compounds as electron donors; however, *“H. subterraneus”* is unable to use H_2_ as an electron donor and appears to be incapable of autotrophic growth (Takai et al., 2001). The genus *Thermocrinis*, which often forms conspicuous streamer growth (Reysenbach et al., 1994; Huber et al., 1998; Eder and Huber, 2002), has only been detected in one 16S rRNA gene census at Rehai (Song et al., 2013) and was not detected by other 16S rRNA gene PCR censuses and phylochip analysis (Pagaling et al., 2012; Hou et al., 2013; Briggs et al., 2014). The sporadic detection of *Thermocrinis* in Rehai may explain why cultivation experiments described here did not yield *Thermocrinis* cultures.

Cultivation-independent surveys in Tengchong also identified abundant *Hydrogenobaculum* populations in Rehai springs with pH < 4, particularly within silica sand-dominated acidic pools in Diretiyanqu and Zhenzuquan (Hou et al., 2013; Song et al., 2013; Briggs et al., 2014). *Hydrogenobaculum* strain T-6, used both H_2_ and reduced S compounds as electron donors (S^2-^, S^0^, and S_2_O_3_^2-^). These compounds are widely used by *Hydrogenobaculum* isolates from different locations (Shima and Suzuki, 1993; Donahoe-Christiansen et al., 2004; D’Imperio et al., 2008), although isolates from Yellowstone National Park are heterogeneous with regard to their ability to oxidize H_2_ (D’Imperio et al., 2008). Arsenite oxidation and the encoding structural genes, *aioBA*, have been documented for some *Hydrogenobaculum* isolates (Donahoe-Christiansen et al., 2004; Clingenpeel et al., 2009; Romano et al., 2013). The *aioBA* genes have also been cloned from natural geothermal environments, including those inhabited by *Hydrogenobaculum* (Clingenpeel et al., 2009; Hamamura et al., 2009) that are similar to those in our study site with regard to temperature and pH. However, strain T-6 was unable to oxidize arsenite under the conditions tested and so perhaps it is similar in this regard to Yellowstone strain Y04AAS1, which lacks recognizable *aioBA* (Romano et al., 2013) and has not been reported to oxidize arsenite. Lack of arsenite oxidation capability may reflect the relatively low concentrations of total arsenic at this site [50–200 ppb (Hou et al., 2013)].

In contrast to the *Aquificaceae*, cultivation-independent surveys have suggested a low abundance of *Hydrogenothermaceae,* including sequences that were related to *Hydrogenothermus*, *Persephonella*, and *Sulfirihydrogenibium* (Pagaling et al., 2012; Song et al., 2013; Briggs et al., 2014). However, two springs not included in published cultivation-independent studies of Rehai yielded strains related to *Sulfurihydrogenibium*. Strain T-7 was very closely related to *S. subterraneum* HGMK-1^T^. The electron donors used by strain T-7 were identical to those used by *S. subterraneum* HGMK-1^T^ (Nakagawa et al., 2005), although T-7 appeared to be more restricted in its use of terminal electron acceptors. Strain T-5 could not grow on any complex organics, and could only use sulfur or thiosulfate as electron donors in the presence of oxygen as an electron acceptor.

## CONCLUSION

This study expands both the geographic and phylogenetic coverage of *Aquificales* cultivated from terrestrial geothermal springs. This study is particularly important within the context of the study of thermophilic microbial communities in Tengchong County because abundant evidence from cultivation-independent studies implicate the *Aquificales* as widely distributed and abundant microorganisms with potential roles in several biogeochemical cycles. Known phenotypic variability within the *Aquificales* notwithstanding, these studies provide a strong foundation for understanding the potential roles of these organisms in C, N, S, and H cycles in the Rehai Geothermal System. The *Aquificales* isolates described here likely represent novel species of *Hydrogenobacter* (strains T-2 and T-8) and *Hydrogenobaculum* (strain T-6) and a new genus in the *Hydrogenothermaceae* (strain T-5). Further work is underway to thoroughly taxonomically describe these novel organisms.

## Conflict of Interest Statement

The authors declare that the research was conducted in the absence of any commercial or financial relationships that could be construed as a potential conflict of interest.
